# Passage of Corpuscles through the Blood-vessels

**Published:** 1871-12

**Authors:** 


PASSAGE OF CORPUSCLES THROUGH THE BLOOD-VESSELS.—A long and important paper on this subject has been lately read before the Royal Society, by Dr. R. Norris, Professor of Physiology in Queen's College, Birmingham. He says that on a careful consideration of the hypotheses which have been propounded by Waller, Cohnheim, Stricker, Bastian, and Caton, to account for the curious phenomena in question, it will be found that all these hypotheses fall short in one important particular, inasmuch as they afford no explanation whatever of by far the most singular part of the process,viz., the fact that the aperatures through which the corpuscles pass again close up and become invisible. The question, indeed, is not so much how the corpuscles get out, as how they get out without leaving any permanent trace of the apertures through which they have so recently passed, and which were so palpable during the period of transit. Before proceeding to elaborate his own views, he restates succinctly the various points upon which observers are agreed. 1st. Both white and red corpuscles pass out of the vessels through aperatures which can neither be seen before their ingress into or egress from the vessel wall, but only during the period of transit. 2nd. An essential and primary step in the process is, that the corpuscles shall adhere or, more properly, cohere to the wall of the vessel. 3rd. These cohering corpuscles shall subsequently be subjected to pressure from within.

From this he proceeds to consider the various physical conditions involved in the question, and eventually he states that all that is essential for a rigid or plastic body to pass through a colloid film is: 1st, an intimate power of cohesion, either mediately or immediately, between the film and the body; 2nd, a certain amount of pressure from within; 3rd, power in the substance of the film to cohere to the surface of the body (or to some intermediate matter which already coheres to the surface) during its passage; 4th, cohesive plasticity of the particles of the material of which the film itself is composed, so that the breach in it may again become reunited as it decends upon the opposite surface of the body which is being extruded. These conditions he thinks are prevalent in the case of the minute blood-vessel and the corpuscle; since he considers that the passage of the corpuscle through the vessel is precisely analogous to the passage of a body through a soap bubble. Unfortunately in parts the author's language is not sufficiently clear to enable one to find his exact meaning, but we believe that we have found it correctly as above. —*London Microscopical Journal.*

